# A novel statistical method to estimate the effective SNP size in vertebrate genomes and categorized genomic regions

**DOI:** 10.1186/1471-2164-7-329

**Published:** 2006-12-29

**Authors:** Daekwan Seo, Cizhong Jiang, Zhongming Zhao

**Affiliations:** 1 Virginia Institute for Psychiatric and Behavioral Genetics, Virginia Commonwealth University, Richmond, VA 23298, USA; 2 Center for the Study of Biological Complexity, Virginia Commonwealth University, Richmond, VA 23284, USA

## Abstract

**Background:**

The local environment of single nucleotide polymorphisms (SNPs) contains abundant genetic information for the study of mechanisms of mutation, genome evolution, and causes of diseases. Recent studies revealed that neighboring-nucleotide biases on SNPs were strong and the genome-wide bias patterns could be represented by a small subset of the total SNPs. It remains unsolved for the estimation of the effective SNP size, the number of SNPs that are sufficient to represent the bias patterns observed from the whole SNP data.

**Results:**

To estimate the effective SNP size, we developed a novel statistical method, SNPKS, which considers both the statistical and biological significances. SNPKS consists of two major steps: to obtain an initial effective size by the Kolmogorov-Smirnov test (KS test) and to find an intermediate effective size by interval evaluation. The SNPKS algorithm was implemented in computer programs and applied to the real SNP data. The effective SNP size was estimated to be 38,200, 39,300, 38,000, and 38,700 in the human, chimpanzee, dog, and mouse genomes, respectively, and 39,100, 39,600, 39,200, and 42,200 in human intergenic, genic, intronic, and CpG island regions, respectively.

**Conclusion:**

SNPKS is the first statistical method to estimate the effective SNP size. It runs efficiently and greatly outperforms the algorithm implemented in SNPNB. The application of SNPKS to the real SNP data revealed the similar small effective SNP size (38,000 – 42,200) in the human, chimpanzee, dog, and mouse genomes as well as in human genomic regions. The findings suggest strong influence of genetic factors across vertebrate genomes.

## Background

Single nucleotide polymorphisms (SNPs) are the most abundant genetic variation in vertebrate genomes. They have been important tools in many biological fields, including mutation mechanisms, genome evolution, disease studies, pharmacogenomics, and fine mapping [1-4]. Strong demands of SNP data and rapid technology advancements helped us to have observed an exponential rate in the discovery of SNPs during the past decade. As of October 2006, the largest public SNP database, dbSNP, deposited more than 87 million submitted SNPs from 35 organisms; among them, more than 50 million SNPs have their references to the genomes [5]. Many more SNPs are to be identified in the near future.

Mutation at the nucleotide level does not occur randomly. Recent studies of mutational mechanisms revealed that the influence of neighboring nucleotides on SNPs was strong in the human and mouse genomes [4,6,7]. Specifically, strong biases relative to the genome average were observed at the two adjacent sites of the SNPs and small biases could extend farther, i.e., as far as 200 nucleotides at each flanking side. Further, the bias patterns varied among the SNP types, e.g., the extent of the biases for transition SNPs (A/G and C/T) was much stronger than those for transversion SNPs (A/C, G/T, A/T, and C/G). Importantly, the bias patterns observed in the whole genome could be sufficiently represented by only a small subset of SNPs randomly sampled from the genome-wide data [8]. The effective SNP size, defined as the minimum number of the SNPs that can essentially represent the bias patterns of the whole SNPs, was roughly estimated to be 30,000 in the human and mouse genomes [8]. Because the SNPs identified in the today's genomes reflect the combinatory evolutionary processes such as methylated CpG mutation hotspots, high transition rate, selection on functional elements, and error-prone DNA replication and repair, a small effective SNP size suggests the strong influence of one or several genetic factors, especially the CpG effects in vertebrate genomes [9,10].

So far, how to efficiently estimate the effective SNP size remains unsolved. The SNPNB, an user-friendly application implemented by Java and Perl, can assist the user to evaluate and obtain a number which is close to the effective SNP size [8]. However, there are three major limitations. First, because SNPNB is based on an empirical re-sampling approach, it becomes impractical to find the effective SNP size when the number of SNPs is very large, which is always true for a genome-wide or chromosome-wide analysis. Second, it is a challenging task on how to define that the bias pattern observed from one data set is (nearly) the same as that from another set. This is because we need to combine four nucleotides at all sites on the 5' side and 3' side of the SNPs. Third, there is a statistical problem. The null hypothesis is that there is no neighboring nucleotide bias of SNPs in the genome, or the frequencies of nucleotides at SNP neighboring sites are the same as the average nucleotide frequencies in the genome sequences. Therefore, the observed neighboring nucleotide biases (%) should be compared to the expected value, which is 0 for each nucleotide at each site. However, a hypothesis test with a very large number of SNPs may not lead to a meaningful conclusion. For example, for the 8,043,656 human SNPs tested in SNPNB [8], when the frequency difference is as low as 0.00028 (0.028%) for nucleotide C, the Z test would be significant at the 5% significance level (α = 0.05). As a result, the null hypothesis is rejected. Obviously, such a small difference is not biologically meaningful or significant. Here, we propose an integrated statistical method to estimate the effective SNP size. This method (SNPKS) considers both the biological significance and the statistical significance so that it avoids the problem of leading to an unreasonably large effective SNP size when only the statistical significance is considered [11]. We also developed an efficient pipeline to iteratively evaluate the intermediate values for the effective size. SNPKS consists of two steps: (1) evaluation of an initial effective size; and (2) iterative tests of the initial effective size by interval evaluation.

## Results

### KS test and interval evaluation

**Figure 1:**
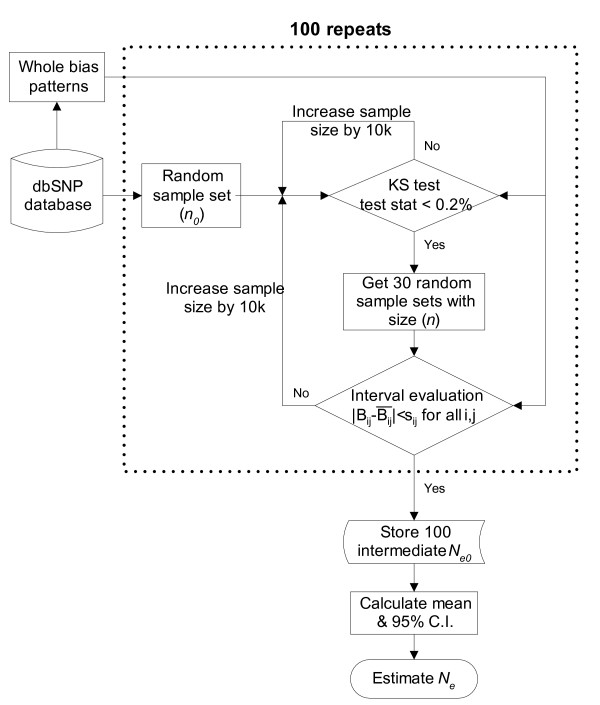
**Flowchart of the SNPKS**. This figure illustrates the integrated two-step procedures in the SNPKS method. KS test: Kolmogorov-Smirnov test; C.I.: confidence interval; *N*_*e*0_: intermediate effective SNP size; *N*_*e*_: effective SNP size.

To estimate the effective SNP size, we designed and integrated a two-step procedure in our system (Figure 1). In the first step, we apply the Kolmogorov-Smirnov test (KS test) [11] to obtain an initial effective SNP size. Usually, the KS test is used to evaluate whether a sample is from a population based on a specific distribution by comparing the corresponding cumulative frequencies. Here we estimate an initial effective SNP size by comparing the cumulative frequencies from the whole SNP data with those from a sample SNP data.

**Figure 2:**
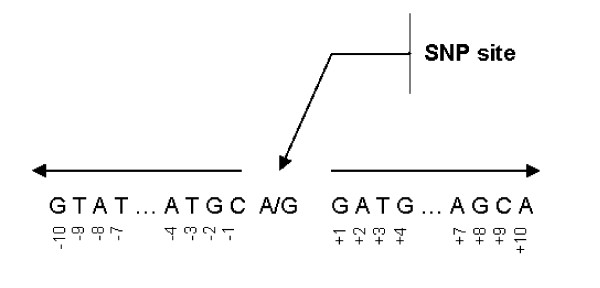
**Annotation of a SNP and its flanking sites**. SNPKS uses ten sites immediately adjacent to the polymorphic site (A/G) at the 5' side and 3' side. A minus sign indicates the flanking site of the 5' side and a positive sign indicates the 3' side.

For the whole SNP data with size *N*, we randomly generate a sub-sample of SNPs with size *n*_0 _(*n*_0_<<*N*). First, we calculate the frequency of each nucleotide at each neighboring site in the whole SNP data and sample SNP data, respectively. According to our previous analyses [6,7], we examine 20 neighboring sites immediately adjacent to each SNP: 10 sites at the 5' side and 10 sites at the 3' side, because these 20 sites have the largest neighboring-nucleotide biases. Figure 2 illustrates the polymorphic site of a SNP and its 5' and 3' flanking sequences. Then, we compare the cumulative relative frequency of each nucleotide in the neighboring sequences. Let *f*_*i*,*j *_and *g*_*i*,*j *_be the frequency of sample SNP data and whole SNP data, respectively, and *F*_*i*,*j *_and *G*_*i*,*j *_be cumulative frequency of sample SNPs and whole SNPs, respectively. Here *i *denotes one of the four nucleotides (A, C, G, and T) and *j *denotes a neighboring site in the SNP flanking sequences (-10 to -1 at the 5' side and +1 to +10 at the 3' side). The *f*_*i*,*j *_and *F*_*i*,*j *_of the sample SNPs are defined by:

fi,j=1n0∑1n0{1if jthnucleotide=i,0otherwise     (1)
 MathType@MTEF@5@5@+=feaafiart1ev1aaatCvAUfKttLearuWrP9MDH5MBPbIqV92AaeXatLxBI9gBaebbnrfifHhDYfgasaacH8akY=wiFfYdH8Gipec8Eeeu0xXdbba9frFj0=OqFfea0dXdd9vqai=hGuQ8kuc9pgc9s8qqaq=dirpe0xb9q8qiLsFr0=vr0=vr0dc8meaabaqaciaacaGaaeqabaqabeGadaaakeaacqWGMbGzdaWgaaWcbaGaemyAaKMaeiilaWIaemOAaOgabeaakiabg2da9maalaaabaGaeGymaedabaGaemOBa42aaSbaaSqaaiabicdaWaqabaaaaOWaaabCaeaadaGabeqaauaabaqaciaaaeaacqaIXaqmaeaacqWGPbqAcqWGMbGzcqqGGaaicqWGQbGAdaahaaWcbeqaaiabdsha0jabdIgaObaakiabd6gaUjabdwha1jabdogaJjabdYgaSjabdwgaLjabd+gaVjabdsha0jabdMgaPjabdsgaKjabdwgaLjabg2da9iabdMgaPjabcYcaSaqaaiabicdaWaqaaiabd+gaVjabdsha0jabdIgaOjabdwgaLjabdkhaYjabdEha3jabdMgaPjabdohaZjabdwgaLbaaaiaawUhaaaWcbaGaeGymaedabaGaemOBa42aaSbaaWqaaiabbcdaWaqabaaaniabggHiLdGccaWLjaGaaCzcamaabmaabaGaeGymaedacaGLOaGaayzkaaaaaa@6825@

Fi,j={fi,−10if j=−10Fi,j−1+fi,jif−9≤j≤10 and j≠0.     (2)
 MathType@MTEF@5@5@+=feaafiart1ev1aaatCvAUfKttLearuWrP9MDH5MBPbIqV92AaeXatLxBI9gBaebbnrfifHhDYfgasaacH8akY=wiFfYdH8Gipec8Eeeu0xXdbba9frFj0=OqFfea0dXdd9vqai=hGuQ8kuc9pgc9s8qqaq=dirpe0xb9q8qiLsFr0=vr0=vr0dc8meaabaqaciaacaGaaeqabaqabeGadaaakeaacqWGgbGrdaWgaaWcbaGaemyAaKMaeiilaWIaemOAaOgabeaakiabg2da9maaceqabaqbaeaabiGaaaqaaiabdAgaMnaaBaaaleaacqWGPbqAcqGGSaalcqGHsislcqaIXaqmcqaIWaamaeqaaaGcbaGaemyAaKMaemOzayMaeeiiaaIaemOAaOMaeyypa0JaeyOeI0IaeGymaeJaeGimaadabaGaemOray0aaSbaaSqaaiabdMgaPjabcYcaSiabdQgaQjabgkHiTiabigdaXaqabaGccqGHRaWkcqWGMbGzdaWgaaWcbaGaemyAaKMaeiilaWIaemOAaOgabeaaaOqaaiabdMgaPjabdAgaMjabgkHiTiabiMda5iabgsMiJkabdQgaQjabgsMiJkabigdaXiabicdaWiabbccaGiabdggaHjabd6gaUjabdsgaKjabbccaGiabdQgaQjabgcMi5kabicdaWaaacqGGUaGlaiaawUhaaiaaxMaacaWLjaWaaeWaaeaacqaIYaGmaiaawIcacaGLPaaaaaa@6940@

The *g*_*i*,*j *_and *G*_*i*,*j *_are defined similarly except for that *N*, the size of the whole SNP data, instead of *n*_0 _is used. The maximum difference of cumulative frequency for each nucleotide is defined by:

Di=max⁡j|Fi,j−Gi,j|     (3)
 MathType@MTEF@5@5@+=feaafiart1ev1aaatCvAUfKttLearuWrP9MDH5MBPbIqV92AaeXatLxBI9gBaebbnrfifHhDYfgasaacH8akY=wiFfYdH8Gipec8Eeeu0xXdbba9frFj0=OqFfea0dXdd9vqai=hGuQ8kuc9pgc9s8qqaq=dirpe0xb9q8qiLsFr0=vr0=vr0dc8meaabaqaciaacaGaaeqabaqabeGadaaakeaacqWGebardaWgaaWcbaGaemyAaKgabeaakiabg2da9maaxababaGagiyBa0MaeiyyaeMaeiiEaGhaleaacqWGQbGAaeqaaOWaaqWaaeaacqWGgbGrdaWgaaWcbaGaemyAaKMaeiilaWIaemOAaOgabeaakiabgkHiTiabdEeahnaaBaaaleaacqWGPbqAcqGGSaalcqWGQbGAaeqaaaGccaGLhWUaayjcSdGaaCzcaiaaxMaadaqadaqaaiabiodaZaGaayjkaiaawMcaaaaa@47B2@

Next, we compare the maximum difference of cumulative relative frequency of each nucleotide with the threshold value of biological significance instead of test statistic given by the KS test, because the tolerable difference in the KS test is too generous to find a reasonable sample size [11]. Here we specify 0.2% as a biological significance threshold value because when the frequency difference is < 0.2%, it appears that the biases are likely due to the stochastic variance and they are not biologically meaningful based on our previous studies [6-8]. When the *D*_*i *_fails to be less than 0.2%, the size of the sample set is increased by 10,000. The procedure above will run it again until the criterion of *D*_*i *_< 0.2% is satisfied (Figure 1). This step gives out an initial effective size (*n*) for the next procedure.

After getting an initial effective SNP size *n*, the second step is to test whether the bias patterns obtained from the sample with this size can effectively represent the bias patterns observed from the whole SNP data. This is performed by an interval evaluation using 30 different SNP subsets with size *n *randomly sampled from the whole SNP dataset. We choose 30 different subsets because when the sample size approaches 30, we can safely assume the distribution of the bias patterns to be normal for inference purpose by central limit theorem. For each nucleotide at each neighboring site, we calculate the bias relative to the genome sequence average (e.g., A: 29.55%, C: 20.44%, G: 20.46%, and T: 29.54% in the human genome) in each of the 30 sample sets, and then get its average bias (Bi,j¯
 MathType@MTEF@5@5@+=feaafiart1ev1aaatCvAUfKttLearuWrP9MDH5MBPbIqV92AaeXatLxBI9gBaebbnrfifHhDYfgasaacH8akY=wiFfYdH8Gipec8Eeeu0xXdbba9frFj0=OqFfea0dXdd9vqai=hGuQ8kuc9pgc9s8qqaq=dirpe0xb9q8qiLsFr0=vr0=vr0dc8meaabaqaciaacaGaaeqabaqabeGadaaakeaadaqdaaqaaiabdkeacnaaBaaaleaacqWGPbqAcqGGSaalcqWGQbGAaeqaaaaaaaa@318E@). When the difference between the average bias in the 30 sample sets (Bi,j¯
 MathType@MTEF@5@5@+=feaafiart1ev1aaatCvAUfKttLearuWrP9MDH5MBPbIqV92AaeXatLxBI9gBaebbnrfifHhDYfgasaacH8akY=wiFfYdH8Gipec8Eeeu0xXdbba9frFj0=OqFfea0dXdd9vqai=hGuQ8kuc9pgc9s8qqaq=dirpe0xb9q8qiLsFr0=vr0=vr0dc8meaabaqaciaacaGaaeqabaqabeGadaaakeaadaqdaaqaaiabdkeacnaaBaaaleaacqWGPbqAcqGGSaalcqWGQbGAaeqaaaaaaaa@318E@) and the corresponding bias in the whole SNP data (*B*_*i*,*j*_) is less than its standard deviation for all nucleotides at all neighboring sites, an intermediate effective SNP size (*N*_*e*0_) is found. That is, the proposed method iteratively evaluates the following difference:

|*B*_*i*,*j *_- Bi,j¯
 MathType@MTEF@5@5@+=feaafiart1ev1aaatCvAUfKttLearuWrP9MDH5MBPbIqV92AaeXatLxBI9gBaebbnrfifHhDYfgasaacH8akY=wiFfYdH8Gipec8Eeeu0xXdbba9frFj0=OqFfea0dXdd9vqai=hGuQ8kuc9pgc9s8qqaq=dirpe0xb9q8qiLsFr0=vr0=vr0dc8meaabaqaciaacaGaaeqabaqabeGadaaakeaadaqdaaqaaiabdkeacnaaBaaaleaacqWGPbqAcqGGSaalcqWGQbGAaeqaaaaaaaa@318E@| <*s*_*i*,*j*_, ∀ *i*, *j *    (4)

where *s*_*i*,*j *_is the standard deviation from the 30 bias patterns. Otherwise, we increase the sample size by 10,000 and run this step again. The procedure runs iteratively until the criterion is satisfied.

The two steps above run repetitively 100 times. This leads to 100 *N*_*e*0 _estimates. The effective SNP size is thus the mean of these 100 estimates.

### Implementation

To implement the SNPKS method, we developed computer programs in C and Perl. In the SNPKS algorithm, we need to regularly generate random numbers and then extract random SNPs from the whole SNP dataset based on the generated random numbers. This routine is computationally intensive; therefore, we wrote a computer program in C. The KS test and interval estimation were implemented in a Perl script, which calls the C program automatically. The application has been tested on both Microsoft Windows and Linux operating systems. The programs, instructions, and test data are available at the website [12].

### Applications

**Figure 3:**
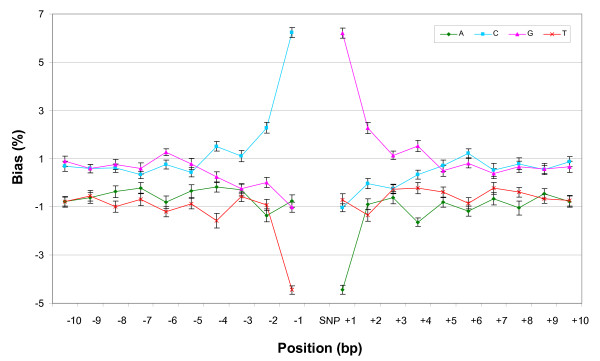
**Neighboring-nucleotide bias patterns for dog SNPs and interval evaluation**. The color lines show the neighboring-nucleotide biases relative to the dog genome sequence average using 2,690,084 dog SNPs. For 30 random sample sets with size 38,000, we obtained their average bias (Bi,j¯
 MathType@MTEF@5@5@+=feaafiart1ev1aaatCvAUfKttLearuWrP9MDH5MBPbIqV92AaeXatLxBI9gBaebbnrfifHhDYfgasaacH8akY=wiFfYdH8Gipec8Eeeu0xXdbba9frFj0=OqFfea0dXdd9vqai=hGuQ8kuc9pgc9s8qqaq=dirpe0xb9q8qiLsFr0=vr0=vr0dc8meaabaqaciaacaGaaeqabaqabeGadaaakeaadaqdaaqaaiabdkeacnaaBaaaleaacqWGPbqAcqGGSaalcqWGQbGAaeqaaaaaaaa@318E@) and standard deviation (*s*_*i*,*j*_) for each nucleotide at each position. The vertical bars represents the interval Bi,j¯
 MathType@MTEF@5@5@+=feaafiart1ev1aaatCvAUfKttLearuWrP9MDH5MBPbIqV92AaeXatLxBI9gBaebbnrfifHhDYfgasaacH8akY=wiFfYdH8Gipec8Eeeu0xXdbba9frFj0=OqFfea0dXdd9vqai=hGuQ8kuc9pgc9s8qqaq=dirpe0xb9q8qiLsFr0=vr0=vr0dc8meaabaqaciaacaGaaeqabaqabeGadaaakeaadaqdaaqaaiabdkeacnaaBaaaleaacqWGPbqAcqGGSaalcqWGQbGAaeqaaaaaaaa@318E@ ± *s*_*i*,*j*_. The figure shows that the intervals at all positions cover the corresponding biases observed from the whole dog SNPs. On the x axis, a minus sign indicates the 5' side and a positive sign indicates the 3' side of the SNPs.

We applied SNPKS to estimate the effective SNP size in four vertebrate genomes: human, chimpanzee, dog, and mouse. The genome-wide SNP data were retrieved from the dbSNP database of the National Center for Biotechnology Information (NCBI) (see Methods). The number of the test SNPs are shown in Table 1. Here we describe the procedures using dog SNPs because there is no previous investigation of the point mutation patterns using SNPs in the dog genome and also our analysis indicated that the neighboring-nucleotide biases were strongest among these four genomes. We started a random sample size 10,000 (*n*_0_) and run the SNPKS programs iteratively. We got an initial effective size (*n*) 38,000. Then, we took 30 random subsets with size 38,000 and performed an interval evaluation. As shown in Figure 3, for all four nucleotides at all 20 neighboring sites, the intervals obtained from the 30 samples covered the frequencies observed from the whole dog SNPs. Table S1 (see Additional file 1) shows the biases relative to the average nucleotide frequencies in dog genome for each neighboring site from whole dog SNPs and from 30 random sample subsets with size 38,000. It also includes the information of standard deviation.

The effective SNP size was estimated to be 38,200 ± 2,500, 39,300 ± 2,100, 38,000 ± 2,300, and 38,700 ± 2,300 for the human, chimpanzee, dog, and mouse genomes, respectively. The 95% confidence intervals were in a narrow range in these four genomes (Table 1). Overall, the effective SNP size (1) is similar in these four genomes, and (2) represents only a small proportion of the genome-wide SNP data (*N*). The comparative results suggest strong genetic influences such as CpG effects across vertebrate genomes [4,13,14].

We next estimated the effective SNP size for the specific genomic regions in the human genome. We used SNPs in intergenic regions, genes, introns, and CpG islands and the average nucleotide frequencies in the corresponding genomic regions. We did not test in exons or untranslated regions (UTRs) because the number of SNPs mapped in exons or UTRs was not sufficient. Although the numbers of SNPs and sequence compositions in the genomic regions varied greatly, their effective SNP sizes were estimated to be similar: 39,100 ± 2,300 (intergenic regions), 39,600 ± 2,200 (genes), 39,200 ± 2,200 (introns), and 42,200 ± 2,700 (CpG islands). The effective SNP size in the CpG islands is the largest. This reflects the lack of methylation and suppression of 5^m^C deamination in CpG islands [15].

### Performance comparison

We compared the performance of our method versus the empirical iterative procedures in SNPNB [8]. We tested on a Dell Workstation (CPU 2 × 3.0 GHz, Memory 4 GB, Redhat Linux Enterprise WS 3.0) using human and mouse SNP data. The results indicated that SNPKS greatly outperformed SNPNB. For human SNP data, SNPNB elapsed ~28 hours by a single round of evaluation and ~151 hours by 10 rounds. This compares to only ~7.5 hours in SNPKS, which doesn't require the recursive rounds to estimate the effective SNP size (Table 2). Assuming that SNPNB requires 10 rounds of evaluation to find a number close to the effective SNP size, it required 20-fold more computation time for human SNP data and 35-fold more time for mouse SNP data than SNPKS (Table 2).

## Discussion

The effective SNP size in this study is defined as the minimum number of SNPs that are sufficient to represent the bias patterns observed from the whole SNP data. It measures the sequence context patterns observed in the SNPs. To our knowledge, this term has not been used in any other report except for our previous study [8]. This term is similar to the effective population size or effective sample size, which has been widely used in the population genetics or disease study. For example, the effective population size (*N*_*e*_) is used to measure the size of an idealized population having the same effect of random sampling on gene frequency as that in the actual population. It can be estimated by *N*_*e *_= θ/4 *μ*, where *θ *is the population parameter and *μ*is the mutation rate per sequence per generation [16]. One example of the effective sample size in SNP analysis (named as the SNP-effective sample size) is to estimate the number of sequences in an alignment given the observed number of SNPs in the sequences [17].

It is important to know how many SNPs are sufficient to represent the bias patterns observed from the whole SNP data. First, this evaluates whether the observed patterns are representative or random in the genome [18,19]. The early studies of mutation pattern often revealed inconsistent results because of their limited size of the data, such as the influence of the neighboring nucleotides on SNPs [4,6,20], mutation direction (e.g., G/C → A/T vs. A/T → G/C) [21-24], and methylation-dependent transition rates [25,26]. To draw a firm conclusion, such an evaluation is required. Second, a small effective size means high confidence of the observed biases and indicates some genetic factors (e.g., CpG effects) that contribute significantly to the biases. Further investigations of these factors will help us understand how mutation occurs in the specific sequence environment and has been maintained or survived during the evolutionary paths. Third, comparative analysis of the bias patterns and effective SNP size should reveal the mutability of the sequences in the different genomic regions among genomes, which is important for the study of genome evolution. Currently, more than 300 genome sequences have been completed and available in NCBI. The comparative genomics is emerging as an important research field. The comparative analysis of SNP data should provide us many insights on the mutability of sequence, genome sequence evolution, genetic drift, and natural selection among different genomes.

In this study, our analysis revealed the similar bias patterns observed in the chimpanzee, dog, human, and mouse genomes. However, the extent of the neighboring-nucleotide biases was different among these four genomes: the strongest in the dog genome and the weakest in the mouse genome (Figure 3, other data not shown). For example, the bias for nucleotide G at the 3' immediate adjacent site was +4.89% (human), +4.80% (chimpanzee), +2.76% (mouse), and +6.21% (dog) relative to the corresponding genome average, respectively. Surprisingly, the effective sizes of the SNPs in these four genomes were similar (Table 1) and not statistically significantly different (ANOVA *P *= 0.83). While this may suggest the strong influence of genetic factors in these genomes, further investigations on these factors and SNP ascertainment biases are warranted. Note that, because the size was increased by 10,000 each time in the iterative procedure in SNPKS, it is unlikely the method led to the similar effective sizes. Further, the effective sizes of the SNPs among the human genomic regions were overall slightly higher than the genome-wide whole SNPs (Table 1). The effective SNP size was the largest in the CpG islands and smallest in the intergenic and intronic regions. This is consistent with the previous findings of the strong CpG effect in the genome except for the CpG islands [13,15] and the possible selection in the CpG islands [27]. Further simulation analysis may figure out how each genetic factor impacts on the effective SNP size. Overall, the sizes obtained in this study were in a small range, 38,000 – 42,200, suggesting that the effective SNP size in vertebrate genomes and their genomic regions is remarkably similar and close to 40,000. This effective size may have three applications. First, it provides a new metric to assess genetic variability in other studies or other genomes. Second, millions of SNPs are to be discovered in many vertebrate genomes in the near future. The effective SNP size may be found larger or smaller than 40,000. Then, comparative genomics studies may uncover one or some genetic factors (e.g., mutability of nucleotides, CpG effect, natural selection, biased gene conversion, recombination, biased DNA mismatch repair) contribute to the difference. Third, it may be used to compare the mutation pattern in a variety of specific datasets, such as disease causing mutations, SNPs with rare allele frequencies or common allele frequencies, different SNP types (e.g., C/T polymorphisms, C → T changes), SNPs at the biased codons or at the fourfold degenerate sites, mutation direction asymmetry at two DNA strands (e.g., A → G vs. T → C).

To examine whether the above estimates of the effective SNP size were reliable, we performed some additional analysis using different datasets. First, SNP discovery and sampling is often subject to ascertainment bias [28]. Some SNPs in the dbSNP database were identified from a very limited number of sequences, even from only two sequences. To examine whether such an ascertainment bias has an effect on the estimation of the effective SNP size, we used HapMap phase I SNPs, which had strong ascertainment bias, and phase II SNPs, which had less sampling ascertainment bias [2]. The effective SNP size was estimated to be similar: 38,400 ± 2,600 for the 861,498 phase I SNPs and 39,100 ± 2,200 for the 2,435,362 phase II SNPs (Table 1). Second, we examined whether a random subset of the total SNPs can have the similar effective size as the total SNPs. We generated 9 random subsets from the human SNPs, with their sizes ranging from 1.0 to 5.0 million. The effective SNP sizes of these 9 subsets were within a range of 36,200 to 39,800 (Table 3) and similar to that (38,200) of the total human SNPs. Third, we compared the effective sizes of two sets of SNPs in the mouse genome: 376,146 SNPs (Build 123) and 7,832,159 (Build 126). Again, the effective sizes were similar (Table 1). These results suggest that the estimation of effective SNP size is less impacted by the ascertainment biases and sample size, thus, is robust.

It is difficult to evaluate whether one bias pattern is statistically the same as another because it needs to compare each of the four nucleotides at each flanking site of SNPs. If we consider 10 neighboring sites at each flanking side of SNPs, we will run and compare 80 multiple statistical tests. It is hard to control type I error (α). If we apply the Bonferroni corrections for 80 multiple comparison tests, we have the significance level α/80 for each test [29]. That means the value of test statistic is too large to lead an unreasonable effective size. Previously, a re-sampling algorithm was implemented to evaluate the effective SNP size [8]. That algorithm is based on an empirical approach and is computationally intensive even with a few rounds of re-sampling when the number of SNPs is large. Moreover, its algorithm can only evaluate a number which is close to the effective SNP size. The SNPKS method proposed in this study first applies the KS test to obtain an initial effective SNP size. Instead of a usual statistical approach, the biological tolerable difference (i.e., 0.2%) is used [11]. This improvement seems robust because the patterns from the sample with size *N*_*e *_are essentially the same as those from the whole SNP data by our visual examination (Figure 3). However, SNPKS is still heuristic. While it should yield adequate approximation of the effective SNP size for practical use, there is no guarantee on finding the absolute minimum effective SNP size.

## Conclusion

We proposed an integrated statistical method (SNPKS) to estimate the effective SNP size. SNPKS consists of two major steps: evaluation of an initial effective size and iterative tests of the size by interval evaluation. SNPKS considers both the biological significance and the statistical significance. SNPKS is the first method to estimate the effective size based on statistical tests and greatly outperforms SNPNB. The application of SNPKS to real SNP data in the human, chimpanzee, dog, and mouse genomes revealed the similar small effective SNP size (i.e., 38,000 – 42,200) in these four genomes and in human genomic regions, suggesting strong influence of genetic factors across vertebrate genomes.

## Methods

### Data preparation

We downloaded SNPs in four vertebrate genomes (chimpanzee, dog, human, and mouse) from the dbSNP database [5]. We retrieved 10,430,753 human SNPs, 1,470,601 chimpanzee SNPs, and 3,023,305 dog SNPs from the Build 125. We retrieved 499,051 mouse SNPs from the Build 123 because we found that more than 1 million SNPs that were newly deposited in the Build 125 were from Perlegen, Inc.. Our analysis indicated that these SNPs were distributed mainly on three chromosomes (2, 4, and 11), which have higher GC content than the mouse genome average. Because we are limited to apply our method to the bias patterns in the whole genome in this analysis, these skewed data would influence the interpretation of the results [7]. When we prepared the manuscript, the dbSNP database released the Build 126, which increased the number of mouse SNPs to 8,274,228. Therefore, we downloaded them for our analysis. We downloaded HapMap SNPs from the International HapMap Project web site [30], including 1,156,867 Phase I SNPs and 3,395,857 Phase II SNPs.

We selected only those SNPs that were biallelic, mapped in the non-repetitive sequences, and at least 20 nucleotides long at each side of flanking sequences. A SNP was assigned in the non-repetitive sequences if the 20 nucleotides at each side of the SNP did not overlap any repeats. A total of 5,200,425 (human), 1,470,501 (chimpanzee), 2,690,084 (dog), 7,832,159 (mouse Build 126), 376,146 (mouse Build 123), 861,498 (HapMap phase I), and 2,435,362 (HapMap phase II) SNPs were extracted after this data process (Table 1). We used these SNPs for SNPKS analysis in this study and named them test SNPs.

We next identified SNPs in human genomic regions using the procedures described in Jiang and Zhao [21]. First, the SNPs in human intergenic, genic, and intronic regions were identified by comparing the SNP locations in the assembled genomic sequences with the coordinates of intergenic regions, genes, and introns. We retrieved the coordinates of each genomic region (e.g., intron) from the ENSEMBL database (version 32.35e, released in July 2005) [31]. We only included the known genic, intronic, and intergenic regions and excluded any genomic region that is predicted or possibly overlapped with another genomic region (e.g., alternative transcripts). We also excluded those SNPs that were not uniquely mapped in the human genome. We identified 2,422,730, 744,987, and 889,956 SNPs in the known intergenic, genic, and intronic regions, respectively (Table 1). Second, we identified SNPs in the CpG islands. CpG islands were identified using the CpG island searching program (CpGi130) [32]. We used stringent search criteria for GC content ≥ 55%, Obs_CpG_/Exp_CpG _≥ 0.65, and length ≥ 500 bp to screen CpG islands in the human genome sequences. The criteria above can effectively exclude the universal Alu repeats, which typically have a sequence length of 300 bp, GC content of 53%, and Obs_CpG_/Exp_CpG _ratio of 0.62 [33,34]. We identified the SNPs in the CpG islands by matching the coordinates of the SNPs with those of the CpG islands in the reference sequences. Again, we excluded the SNPs that were not uniquely mapped in the human genome. A total of 95,561 SNPs were identified in the human CpG islands.

## Authors' contributions

DS participated in the data preparation and the method development, carried out computational work, and helped to draft the manuscript. CJ participated in the data preparation and analysis. ZZ conceived of the study, participated in its design, method development, and coordination, and helped to draft the manuscript. All authors read and approved the final manuscript.

## Supplementary Material

Additional file 1 Bias comparison using genome-wide dog SNPs. Supplementary Table S1 – Bias comparison using genome-wide dog SNPs. Click here for file

## Figures and Tables

**Table 1:** Estimation of the effective SNP size

	Total # of test SNPs	Effective SNP size (*N*_*e*_)	95% C.I.
Human	5,200,425	38,200	35,700 – 40,700
Chimpanzee	1,470,501	39,300	37,200 – 41,400
Dog	2,690,084	38,000	35,700 – 40,300
Mouse (Build 126)	7,832,159	38,700	36,400 – 41,000
Mouse (Build 123)	376,146	39,100	36,800 – 41,400
			
Human HapMap phase I	861,498	38,400	35,800 – 41,000
Human HapMap phase II	2,435,362	39,100	36,900 – 41,300
			
Human intergenic region^a^	2,422,730	39,100	36,800 – 41,400
Human gene^a^	744,987	39,600	37,400 – 41,800
Human intron^a^	889,956	39,200	37,000 – 41,400
Human CpG island^a^	95,561	42,200	39,500 – 44,900

^a^The average nucleotide frequencies in the genomic regions were used.

**Table 2:** Performance comparison with SNPNB

SNP data	Process^a^	SNPNB [8]			SNPKS
			
		1 round	5 rounds	10 rounds	
Human	Preprocessing data	2 h 50 m 25 s			2 h 24 m 49 s
	Estimation of *N*_*e*_	24 h 56 m 1 s	82 h 48 m 1 s	147 h 39 m 40 s	5 h 7 m 35 s
	Total elapsed time	27 h 46 m 26 s	85 h 38 m 26 s	151 h 8 m 56 s	7 h 32 m 24 s
					
Mouse (Build 123)	Preprocessing data	0 h 2 m 51 s			0 h 2 m 52 s
	Estimation of *N*_*e*_	7 h 27 m 55 s	37 h 53 m 53 s	75 h 18 m 28 s	2 h 4 m 50 s
	Total elapsed time	7 h 30 m 46 s	37 h 56 m 44 s	75 h 21 m 19 s	2 h 7 m 42 s

^a^The tests were performed in a Linux workstation (CPU 2 × 3.0 GHz, memory 4 GB).

**Table 3:** Estimation of the effective SNP size in random subsets of human SNPs

Sample size	Effective SNP size (*N*_*e*_)	95% C.I.
1.0 × 10^6^	37,000	34,700 – 39,300
1.5 × 10^6^	39,600	37,200 – 42,000
2.0 × 10^6^	38,600	36,000 – 41,200
2.5 × 10^6^	38,100	35,800 – 40,400
3.0 × 10^6^	36,200	33,700 – 38,700
3.5 × 10^6^	38,600	36,500 – 40,700
4.0 × 10^6^	37,300	35,000 – 39,600
4.5 × 10^6^	39,200	36,900 – 41,500
5.0 × 10^6^	39,800	37,500 – 42,100

The effective SNP sizes of these 9 subsets are not statistically significantly different (ANOVA, *P *= 0.40).
