# Supplementary material for: A novel statistical method to estimate the effective SNP size in vertebrate genomes and categorized genomic regions
Source: BMC Genomics. 2006 Dec 29;7:329. doi: 10.1186/1471-2164-7-329 (PMC1769377; doi:10.1186/1471-2164-7-329)
Supplement: Additional file 1 — Bias comparison using genome-wide dog SNPs. Supplementary Table S1 – Bias comparison using genome-wide dog SNPs. [file 1471-2164-7-329-S1.doc]

## Table S1 - Bias comparison using genome-wide dog SNPs

^a^Position relative to the polymorphic site. A minus sign indicates the 5’ side and a positive sign indicates the 3’ side.
